# Patients receiving opioid maintenance treatment in primary care: successful chronic hepatitis C care in a real world setting

**DOI:** 10.1186/1471-2334-13-9

**Published:** 2013-01-08

**Authors:** André Seidenberg, Thomas Rosemann, Oliver Senn

**Affiliations:** 1 Institute of General Practice and Health Services Research, University of Zurich, Zurich, Switzerland; 2 General Private Practice, Zurich, Switzerland

**Keywords:** Hepatitis C, Methadone substitution, Primary care

## Abstract

**Background:**

Injection drug users (IDUs) represent a significant proportion of patients with chronic hepatitis C (CHC). The low treatment uptake among these patients results in a low treatment effectiveness and a limited public health impact. We hypothesised that a general practitioner (GP) providing an opioid maintenance treatment (OMT) for addicted patients can achieve CHC treatment and sustained virological response rates (SVR) comparable to patients without drug dependency.

**Methods:**

Retrospective patient record analysis of 85 CHC patients who received OMT for more than 3 months in a single-handed general practice in Zurich from January 1, 2002 through May 31, 2008. CHC treatment was based on a combination with pegylated interferon and ribavirin. Treatment uptake and SVR (undetectable HCV RNA 6 months after end of treatment) were assessed. The association between treatment uptake and patient characteristics was investigated by multiple logistic regression.

**Results:**

In 35 out of 85 CHC patients (52 males) with a median (IQR) age of 38.8 (35.0-44.4) years, antiviral therapy was started (41.2%). Median duration (IQR) of OMT in the treatment group was 55.0 (35.0-110.1) months compared to the group without therapy 24.0 (9.8-46.3) months (p<0.001). OMT duration remained a significant determinant for treatment uptake when controlled for potential confounding. SVR was achieved in 25 out of 35 patients (71%).

**Conclusion:**

In addicted patients a high CHC treatment and viral eradication rate in a primary care setting in Switzerland is feasible. Opioid substitution seems a beneficial framework for CHC care in this “difficult to treat” population.

## Background

Chronic hepatitis C infection (CHC) is a major cause of end-stage liver disease (ESLD). Injection drug users (IDUs) are a major risk group for HCV infection [1] thus they significantly contribute to the patient group with ESLD in the future [2]. In recent years treatment efficacy of CHC has been steadily increased and a successful virus eradication can be achieved in up to 80% of patients with genotype 2 and 3 and in about 50% of patients chronically infected with genotype 1 [3-5]. However data suggest that about 70% of patients with confirmed CHC go untreated [6-8] therefore reducing therapeutic effectiveness of an anti-HCV therapy.

Increasing evidence suggests similar treatment efficacy of a pegylated interferon-based (pegIFN) combination therapy with ribavirin on HCV clearance in patients with substance abuse compared to patients without drug dependency [9-13]. Despite the revised NIH consensus on medical management of HCV infection that recommended a treatment decision on an individual basis [14] current clinical practice suggests that CHC patients with a history of IDU have a lower chance of getting antiviral therapy compared to other patient groups [7,15]. This further emphasize that the true therapeutic impact of an anti-CHC therapy is rather an issue of access to treatment than of drug efficacy.

In IDUs concomitant illicit drug use, problem drinking, comorbid medical (e.g. HIV) and psychiatric conditions are prevalent representing common barriers to antiviral treatment. On the other hand opioid maintenance treatment (OMT) is established in the therapy of substance abuse [16] and has been shown to be an effective framework for adherence and virological success in HIV treatment [17,18]. In addition we previously demonstrated the beneficial role of a substitution treatment as a therapeutic framework for a successful CHC case finding [19]. We hypothesized that a general practitioner (GP) providing an integrated chronic care approach including an OMT can achieve similar CHC treatment rates and viral eradication rates compared to patients without drug dependency. In addition we aimed to assess patient characteristics including duration of OMT which were associated with CHC treatment initiation in a primary care setting.

## Methods

### Patients and setting

Retrospective records review of patients receiving opioid maintenance treatment (OMT) in the study period between January 1, 2002 through May 31, 2008. A detailed description of the study population and the setting has been previously reported elsewhere [19]. Briefly, all patients ≥ 18 years old and participating for at least 3 months in an official office-based OMT programme run by a single-handed general practice located in Zurich, Switzerland were eligible for CHC assessment and evaluation for antiviral therapy in the case of a CHC infection. In 327 out of 360 patients (90.8%) on OMT a successful CHC assessment could be performed and 85 out of the 327 patients (26%) were chronically infected with hepatitis C. The OMT regimen (methadone, buprenorphine or morphine) was based on a computer-assisted drug prescription and delivery system (CDDD) that provides a patient tailored dosage regime within a safety framework allowing the patients to choose dosage within an individualised maximal daily dose [20]. The diagnosis of chronic hepatitis C was based on a detectable HCV RNA concentration and a reported HCV genotype performed by an approved laboratory using a quantitative polymerase-chain reaction. All patients had not been treated previously for CHC. The practice staff has long experience and is specifically trained in OMT and HCV care. All HCV treatments were given in the general practice coordinated and supervised by the GP. A close collaboration within a network of hepatologists, infectiologists and psychiatrists guaranteed rapid access to spezialized care within less than three days. Prior to the OMT and HCV screening patients were asked for their informed consent in an anonymous data analysis. Under Swiss ethics guidelines the study did not require a formal ethics approval.

### Treatment protocol

In all 85 CHC patients risks and opportunities of a specific antiviral therapy have been discussed. Patients infected with HCV genotypes 1 and 4 were treated with once-weekly injections of peginterferon alfa-2a (180 μg), plus ribavirin (1000 mg or 1200 mg/day by weight in divided dose) for 48 weeks. A treatment regimen of ribavirin 800 mg/day (divided in two doses) and peginterferon alfa-2a 180 μg/week subcutaneously for 24 weeks was used for patients with HCV genotype 3 (no patient of the current study population was infected with genotype 2). Peginterferon injections were applied by the medical staff of the general practice mostly while patients received their substitution treatment. Direct dose adjustments according to current treatment guidelines were possible as laboratory point-of care testing (differential blood count) was available at the general practice. Ribavirin has been distributed on a weekly basis in patients with good compliance and a daytime structure (e.g. certificate of employment) or on a daily observed treatment regimen. Viremia was measured at week 12, at the end of treatment, and 6 months after the end of treatment.

Prior to antiviral treatment start patients with poorly controlled psychiatric disorders were stabilized by pharmacological therapies. A prophylactic treatment was started in patients with a history of depressive disorder before beginning an antiviral therapy. Patients were systematically counselled for a reliable contraception and in women a hormone-releasing intrauterine device (Mirena™) was routinely recommended. Substance abuse including injection drug use was not an exclusion criteria for an interferon-based therapy, however abstinence (or at least a substantial reduction) of alcohol and concomitant substance abuse was strongly recommended in all patients.

### Outcome measures and assessments

Treatment rate and treatment success were primary outcomes and defined as follow: The proportion of evaluated CHC patients who started an interferon-based therapy and the proportion of treated patients with a sustained virologic response (SVR) (i.e. undetectable HCV RNA in the serum 6 months after end of treatment). Viral eradication rate at the end of treatment (ETR) and SVR according to genotypes and in HIV-coinfected patients were secondary outcomes.

OMT-related data (i.e. duration, average dose) could be accessed and further processed from CDDD (see above). Demographics, psychiatric co-morbidities (based on clinical grounds), self-declared addiction-specific variables (i.e. history of IDU and current IDU, concomitant problematic drug and alcohol abuse), and social- and occupational characteristics (i.e. housing situation, employability expressed as a percentage of a full time aequivalent based on a certificate of employment) were assessed by systematic charts review. Problematic substance misuse including alcohol was defined on consensus within the practice team based on clinical observation and patients’ self-reports during routine daily or weekly OMT provision. Substance users’ self-reports have been shown to be reliable, especially in OMT programmes that assure confidentiality and absence of adverse consequences [21]. Patients suffering from a severe adverse event related to a psychiatric co-morbidity (e.g. suicidal behaviour, psychiatric hospitalisation) were considered as unstable.

### Statistical analysis

Descriptive statistics were calculated for all variables. Patients were categorized according to their history of CHC treatment (i.e. “treatment start” vs. “no CHC treatment”). Non-parametric group comparisons were performed to test for differences in distributions of patient characteristics. A multiple logistic regression was used to further assess the independent associations between CHC treatment history and patient characteristics. All statistical analysis were performed using STATA for Windows (version 10.1; Stata Corp., College Station, Texas).

## Results

Baseline characteristics of the 85 patients receiving OMT with a concomitant diagnosis of chronic hepatitis C (CHC) are described in Table 1. Patients started substance abuse on average at the age (IQR) of 18.6 (15.6-20.7) years. Median (IQR) age and age entering the current OMT programme was 38.8 (35.0-44.4) and 34.0 (30.2-39.6) years, respectively. A vast majority of the patients (90.6%) was on methadone maintenance treatment and 11 patients (12.9%) experienced more than 1 opiate for long-term substitution. The median (IQR) duration on OMT was 37.1 (14.5-72) months with a wide range between 4 to 310.9 months. During the study period median prescription for methadone, buprenorphine and morphine was 72.8 (50.1-119.8), 6.2 (3.0-6.5), and 430.5 (312.1-514.8) milligram, respectively. Psychiatric comorbidity was high with about 40% suffering from an unstable disorder and almost one third having a stable condition with or without treatment. The majority of the patients (70.3%) suffering from an unstable psychiatric disorder could have been referred for a psychiatric treatment. All of the referred patients have been diagnosed with a mood disorder and the majority (80.8%) of them suffered from a psychiatric comorbide personality disorder. Former injection drug use (IDU) was reported in the vast majority of the patients and almost 20% reported current IDU. Problematic drug abuse or excessive alcohol consumption was present in almost one third of the patients. Most of the patients had a permanent residence with part- or fulltime employment. HIV-co-infection prevalence was 20%.

**Table 1:** Characteristics of CHC patients >3 months on opioid maintenance treatment (OMT)

**Demographics**	% (n)* or median [IQR] (n)*
Male	61.2 (52)
Age (years)	38.8 (35.0-44.4)
Opioid maintenance therapy (OMT):	
Age beginning substance abuse (years)	18.3 (15.6-20.7)
Age beginning the current OMT (years)	34.0 (30.2-39.6)
Methadone as last OMT medication	90.6 (77)
Buprenorphin as last OMT medication	4.7 [4]
Morphin as last OMT medication	4.7 [4]
Use of ≥1 different opioid in the past	12.9 [11]
Duration of OMT (months)	37.1 (14.5-72)
Average methadone dose during OMT (mg)	72.8 (50.1-119.8)
Psychiatric comorbidity:	
No psychiatric disorder	28.2 [24]
Stable disorder without treatment	12.9 [11]
Stable disorder under treatment	15.3 [13]
Unstable disorder	43.5 (37)
Injection drug use (IDU):	
In the past	92.9 (79)
Current	17.7 [15]
Other drug and excessive alcohol use:	
Heroin	27.1 [23]
Cocaine	25.9 [22]
Benzodiazepines	20.2 [17]
Alcohol	31.8 [27]
Occupational and housing variables:	
Employability (%)	60 (30–100)
Single household	54.1 (46)
Shared household	36.5 (31)
Other lodging ^#^	9.4 [8]
HIV-co-infection	
HIV positive	20 [16]*

*Total number of patients= 85; HIV status was missing in 5 patients; ^#^lodging of patients without stable housing was as follows: homeless [2], asylum [2], social institutions [2], accommodation by friends [2].

### CHC treatment rate and patient determinants for treatment decision

In 35 patients out of the 85 CHC patients an antiviral treatment with pegylated interferon and ribavirin was started, corresponding to a treatment rate of 41.2%. Group comparisons between patients who started a therapy and patients who disagreed to a HCV treatment are presented in Table 2. Duration of OMT was significantly longer in patients who started an antiviral therapy. This association remained statistically significant and revealed an odds ratio (95% CI) of 1.020 (1.006-1.04) (p=0.01) when controlling for potential confounders in a multiple logistic regression. There was no evidence of a non-linear relationship between OMT duration and treatment uptake. The most frequent reason against CHC treatment was patient refusal in 40 cases (80%). No treatment was performed due to various somatic reasons in the remaining 10 patients (20%).

**Table 2:** Determinants of CHC treatment decision in CHC patients >3 months on OMT

**Determinants**	**Treatment start (n=35)**	**No CHC treatment (n=50)**	P-value*
	**% (n) or median [IQR]**	**% (n) or median [IQR]**	
Demographics:			
Male	65.7 [23]	58 [29]	
Female	34.3 [12]	42 [21]	0.48
Age (years)	41.8 (37.2-45.1)	37.5 (34.8-43.8)	0.06
Age beginning OMT (years)	35.0 (31.9-39.9)	33.4 (29.2-39.1)	0.45
Opiate maintenance therapy:			
Methadone	88.5 (31)	92 (46)	
Buprenorphin	8.6 [3]	2 [1]	
Morphin	2.9 [1]	6 [3]	0.51
Single OMT therapy	88.6 (31)	86 (43)	
Use of ≥1 different opiate in the past	11.4 [4]	14 [7]	1.0
Duration of OMT (months)	55.0 (35.0-110.1)	24.0 (9.8-46.3)	<0.001^#^
Average methadone dose during OMT (mg)	73 (50–120)	73 (50–122)	0.83
Psychiatric comorbidity:			
None or stable disorder	68.6 [24]	52 [24]	
Unstable disorder	31.4 [11]	48 [26]	0.060
Injection drug use (IDU):			
In the past, yes	91 (32)	94 (47)	
In the past, no	9 [3]	6 [3]	0.69
Current, yes	8.6 [3]	24 [12]	
Current, no	91.4 (32)	76 (38)	0.086
Other drug and excessive alcohol use:			
Current heroin use	20 [7]	32 [16]	
No heroin use	80 [28]	68 (34)	0.22
Current cocaine use	25.7 [9]	26 [13]	
No cocaine use	74.3 [26]	74 (37)	0.98
Current benzodiazepine use	14.7 [5]	26 [12]	
No benzodiazepine use	85.3 [29]	74 (38)	0.41
Excessive alcohol use	28.6 [10]	34 [17]	
No alcohol excess	71.4 [25]	66 (33)	0.60
Occupational and housing variables:			
Employability (%)	60 (30–100)	60 (30–100)	0.52
Single household	40 [14]	34 [17]	
Shared household	51.4 [18]	56 [28]	
Other lodging	8.6 [3]	10 [5]	0.90
HIV-co-infection^†^			
HIV positive	14.7 [5]	23.9 [11]	
HIV negative	85.3 [29]	76.1 (35)	0.40

*Group comparisons by Chi-square or Fisher exact as appropriate and Mann–Whitney test;

^#^ remained independently associated with treatment start after controlling for age, sex, psychiatric comorbidity, IDU, drug and alcohol use, employability, housing and HIV-co-infection with an OR (95% CI) of 1.02 (1.006-1.04) (p=0.01); ^†^HIV-status was missing in 5 patients.

### CHC treatment success

In 25 out of 35 patients who started a CHC treatment no HCV-RNA could be detected 6 months after the end of treatment corresponding to a sustained virological response rate (SVR) of 71%. Three patients with successful HCV-clearance after initial treatment suffered from a relapse, corresponding to a success rate at the end of treatment of 80% (28/35). When stratified according to HCV genotypes SVR was 76.% in genotype 3 (10 out of 13 patients), 73.7% in genotype 1 (14 out of 19 patients), and 33.3% in genotype 4 (1 out of 3 patients). In 3 out of the 5 HIV-co-infected patients a SVR could be achieved.

Reasons for a failure of pegIFN+RBV in the 10 patients starting an antiviral therapy were as follow: non-responder (n=6), relapse (n=3), early treatment stop due to medical reasons (n=1).

## Discussion

This study examined CHC treatment and success rates in unselected patients appearing “difficult to treat” in a single-handed general practice in Switzerland. Although a substantial proportion of the study population suffered from psychiatric comorbidities, reported excessive alcohol consumption and current drug misuse including injection drug use, treatment could be started in 41.2% and resulted in an overall sustained virological response rate of 71%. The duration of OMT was associated independently and positively with the start of a CHC treatment.

The observed treatment rate compares favourably with previous studies although comparisons have to be interpreted with caution as patient populations differ greatly across studies. In a UK population of CHC patients, Irving et al. [22] reported an overall treatment rate of 10.2% with a wide range varying according to the source of original HCV screening test with the highest rate of 21.4% in patients referred from GPs and a treatment rate of only 1.6% in patients originally screened for HCV by specialist units for drug and alcohol. Butt and colleagues [23] found a treatment rate of 11.8% in unselected veterans who were older (median age 50 years) compared to our study sample but comparable with regard to the prevalence of comorbidities such as psychiatric disorders and drug and alcohol dependence. The cumulative chance of starting CHC treatment in a 5 year period has been estimated to 33% in a representative Danish CHC cohort study [24]. Among patients attending the specialist consultations of the Swiss Hepatitis C Cohort Study (SDDS) [25] a history of CHC related treatment has been reported in 31% of the whole cohort and in 51% in the subgroup of cirrhotics. We found an independent and positive association between treatment initiation and the duration of OMT. Thus a patient spending 37 months (i.e. the median duration of OMT in our study population) on opioid substitution has doubled the chance ( i.e. OR 1.02^37 months^ =2.0) of getting started CHC treatment compared to a patient simply fulfilling the inclusion criteria of minimum 3 months on substitution. This beneficial effect of OMT has already been shown to increase adherence and virological success in HIV treatment [16,18] and is in line with the finding that an ongoing OMT significantly increased the chance of a successful CHC case finding [19]. We are not aware of a study that has assessed the specific role of an OMT programme in the context of CHC treatment initiation. Our results further strengthened OMT as a favourable therapeutic setting, expanding its role for HCV-related care.

We achieved a viral eradication rate similar compared to randomized controlled efficacy trials reporting a sustained virological response up to 80% of patients with genotype 2 and 3 and in about 50% of patients chronically infected with genotype 1 [3-5]. The selection criteria for the aforementioned multicenter registration trials however excluded IDUs thus although internal validity of these studies is high illicit drug users constitute the largest proportion of CHC patients and the results do not represent treatment effectiveness in a “real world setting”. Our response rate is in line with a recent meta-analysis suggesting similar treatment efficacy of an antiviral therapy on HCV clearance in patients receiving OMT compared to patients without drug dependency [9]. However in only 3 out of the 16 studies included in the aforementioned meta-analysis active ongoing illicit drug use was not an exclusion criteria thus our results provided further evidence that successful viral eradication is feasible in a population normally excluded from clinical trials and judged “difficult to treat”.

We are aware that the analysis of our study does not address the CHC treatment effectiveness of a primary care setting in comparison to a specialized medical care setting due to the lack of a randomised controlled intervention. The current standard of care for the treatment of chronic HCV infection which is also recommended for patients with substance abuse was applied by the attending GP with a high level of commitment and a special interest in addiction medicine. Providing chronic care (i.e. substitution, psychiatric comorbidities) in combination with acute somatic care is a main feature of a primary care setting. Our treatment rate is in line with a recent randomized controlled study evaluating the impact of an integrated care approach among CHC patients originally deferred from CHC therapy due to mental health and substance abuse comorbidities [26]. In this study patients receiving an integrated care intervention reached a treatment eligibility of 42% compared to the standard care group with a significant lower eligibility rate of 18%. In our population the level of homelessness and unemployment was relatively low but similar when compared to GP patients in other OMT programmes in Switzerland, which probably reflects the low-threshold management of drug addicts in Switzerland [27]. Furthermore it is important to note that an OMT durationof fewer than 3 months was an exclusion criteria thus a selection of our population probably related to a high level of integration in the social framework has to be considered when compared to other settings.

From a clinical perspective the development of new direct acting antiviral agents such as the protease inhibitors boceprevir and telaprevir are long-awaited and their implementation to clinical practice in the near future have the potential for a new standard of care for the treatment of chronic hepatitis C [28]. Although these new antiviral agents showed superior cure rates compared to the current standard of care the public health impact of these new and better medications will remain limited unless more patients are diagnosed and treatment is initiated [29,30]. To control chronic hepatitis C an increase in treatment uptake is crucial especially in the population of (former) IDUs as these patients have by far the highest prevalence of hepatitis C. On the other hand barriers to HCV-related care are most likely in this “hard to reach” population. An easy to access antiviral treatment linked to OMT has the potential to optimize treatment and cure rates in this “hard to reach” population. We provided a successful example of a low-threshold HCV-related care in a Swiss primary care setting.

## Conclusions

We conclude that a high rate of CHC eradication in unselected patients on OMT is feasible in a primary care setting in Switzerland. The independent association between OMT duration and treatment initiation highlights the important role of opioid substitution as a therapeutic framework to optimize CHC care in a patient group that significantly contributes to the global burden of liver diseases caused by HCV infection.

## Abbreviations

CHC: Chronic hepatitis C; IDU: Injection drug user; GP: General practitioner; SVR: Sustained virological response; OMT: Opioid maintenance treatment; pegIFN: Pegylated interferon.

## Competing interest

The authors declare that they have no competing interest.

## Authors’ contributions

Drs. Seidenberg, Rosemann and Senn designed the study. Dr. Seidenberg collected data and was responsible physician for the opioid maintenance program and the hepatitis C treatment. Drs. Senn and Seidenberg managed the literature searches and summaries of previous related works. Dr. Senn undertook the statistical analysis and wrote the first draft of the manuscript, which was revised and edited by all authors. All authors contributed to and approved the final manuscript.

## Pre-publication history

The pre-publication history for this paper can be accessed here:

http://www.biomedcentral.com/1471-2334/13/9/prepub
